# MutSpec: a Galaxy toolbox for streamlined analyses of somatic mutation spectra in human and mouse cancer genomes

**DOI:** 10.1186/s12859-016-1011-z

**Published:** 2016-04-18

**Authors:** Maude Ardin, Vincent Cahais, Xavier Castells, Liacine Bouaoun, Graham Byrnes, Zdenko Herceg, Jiri Zavadil, Magali Olivier

**Affiliations:** Molecular Mechanisms and Biomarkers Group, International Agency for Research on Cancer, F69372 Lyon, France; Epigenetic Group, International Agency for Research on Cancer, F69372 Lyon, France; Biostatistics Group, International Agency for Research on Cancer, F69372 Lyon, France

**Keywords:** Galaxy, Mutation spectra, Mutation signatures, Single base substitutions

## Abstract

**Background:**

The nature of somatic mutations observed in human tumors at single gene or genome-wide levels can reveal information on past carcinogenic exposures and mutational processes contributing to tumor development. While large amounts of sequencing data are being generated, the associated analysis and interpretation of mutation patterns that may reveal clues about the natural history of cancer present complex and challenging tasks that require advanced bioinformatics skills. To make such analyses accessible to a wider community of researchers with no programming expertise, we have developed within the web-based user-friendly platform Galaxy a first-of-its-kind package called MutSpec.

**Results:**

MutSpec includes a set of tools that perform variant annotation and use advanced statistics for the identification of mutation signatures present in cancer genomes and for comparing the obtained signatures with those published in the COSMIC database and other sources. MutSpec offers an accessible framework for building reproducible analysis pipelines, integrating existing methods and scripts developed in-house with publicly available R packages. MutSpec may be used to analyse data from whole-exome, whole-genome or targeted sequencing experiments performed on human or mouse genomes. Results are provided in various formats including rich graphical outputs. An example is presented to illustrate the package functionalities, the straightforward workflow analysis and the richness of the statistics and publication-grade graphics produced by the tool.

**Conclusions:**

MutSpec offers an easy-to-use graphical interface embedded in the popular Galaxy platform that can be used by researchers with limited programming or bioinformatics expertise to analyse mutation signatures present in cancer genomes. MutSpec can thus effectively assist in the discovery of complex mutational processes resulting from exogenous and endogenous carcinogenic insults.

**Electronic supplementary material:**

The online version of this article (doi:10.1186/s12859-016-1011-z) contains supplementary material, which is available to authorized users.

## Background

DNA mutations accumulate during the natural history of tumors, reflecting the insults from endogenous and exogenous mutagenic processes as well as the selection of cancer-driving events. The nature of somatic mutations observed in single genes or on a genome-wide scale in human tumors can thus reveal information on past carcinogenic exposures and provide clues on cancer etiology [1, 2]. Current efforts in the systematic sequencing of tumor genomes generate large amounts of data on mutation patterns that characterise human cancers. Recent analyses of these data have revealed over 30 somatic mutation signatures [1, 3–6]. While suspected mutational processes have been proposed for some of these signatures, the majority have not yet been attributed to any specific mechanism and their origins thus remain unexplained. Experimental systems developed for modelling in vitro and in vivo genome-wide mutational processes have been reported recently [7–13]. These assays have the potential to generate direct evidence for the identification of carcinogens or mutagenic processes underlying the mutation signatures observed in human tumors. The analysis of experimental and human-derived data requires advanced bioinformatics skills and thus remains limited to a small research community. Tools that would allow streamlined analyses of mutation spectra from genome-wide sequencing data and be accessible to a wider community could speed up research in this area.

Galaxy is a web-based platform that allows the integration of complex programs or scripts built in any language and accessible in a single web interface [14–16]. Tools can be built so that users with no programming skills can perform complex analyses through a user-friendly graphical interface.

Here we present a set of Galaxy tools named MutSpec that offer an accessible framework for advanced analyses of mutation spectra and signatures present in human cancers or experimental systems. MutSpec expands on existing approaches and methods and integrates scripts developed in-house with publicly available R packages to offer a user-friendly interface accessible to biologists with no or limited bioinformatics skills. MutSpec is expected to accelerate the interpretation of mutation patterns observed in human cancers by facilitating their analysis by a wider community and should thus contribute to the identification of new human carcinogens and to a better understanding of how these carcinogens impact the genome.

## Implementation

### Overview and code sources

**Fig. 1:**
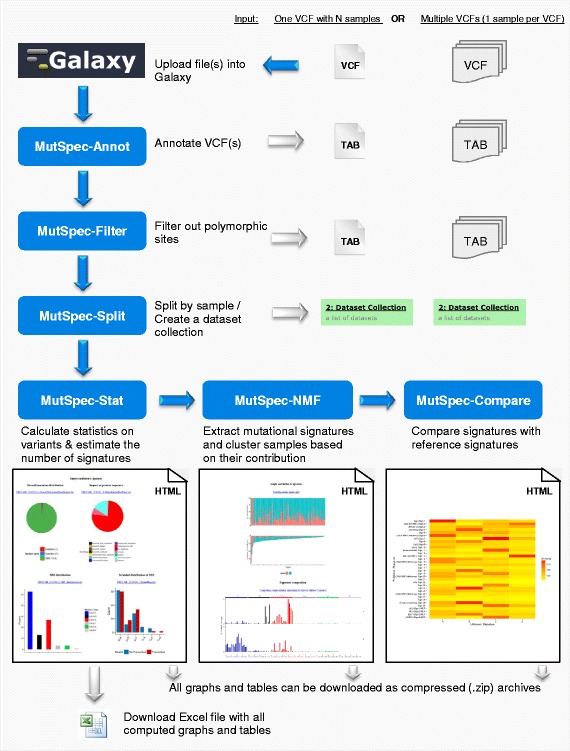
Overview of MutSpec tools and workflow. List of variants identified in a set of cancer samples may be imported as a single VCF files that contains all samples (identified by a sample ID) or as multiple VCFs (one for each sample). The first tool to use is MutSpec_Annot for annotating variants with structural and functional information. These annotations may be used to filter out variants that are known polymorphisms or located in segmental duplication regions with the MutSpec-Filter tool. If a single VCF file containing several samples is uploaded, the MutSpec-Split tool should be used to split data by sample using the sample ID. This tool generates automatically a dataset collection. If multiple VCFs are uploaded, MutSpec-Split should not be run but the annotated VCF should be grouped in a dataset collection. MutSpec-Stat can then be run on the dataset collection to generate various statistics on variants characteristics. These statistics may be visualized as graphs on html pages or downloaded as a single Excel file. The report generated by MutSpec-Stat can then be used as input of MutSpec-NMF to extract mutation signatures present in the sample set. MutSpec-NMF generates plots showing the identified signatures and the contribution of each signature to the mutation load of each sample. Finally, MutSpec-Compare can be used to calculate cosine similarity values between the obtained signatures and a set of reference signatures (published or user-defined). These results are shown as a heatmap

MutSpec is an implementation of Perl and R scripts or packages into several Galaxy tools designed to (1) annotate genome variations (MutSpec-Annot), (2) filter and parse list of variants (MutSpec-Filter and MutSpec-Split respectively), (3) compute various statistics describing mutation spectra features (MutSpec-Stat), (4) extract mutation signatures defined by the six types of single base substitutions (SBS) in their trinucleotide sequence context (MutSpec-NMF), (5) compare the obtained signatures with published ones (MutSpec-Compare). The tools are designed to work in a logical sequence, using as input the outputs of each preceding tool. A typical analysis workflow is shown in Fig. 1. The public packages used and the Perl scripts that support each tool are described in Table 1. The tools produce simple tab-delimited text files or content-rich html pages with graphical representations of the data and hyperlinks to underlying data. All figures and tables that are produced by the different tools can be downloaded as individual files. Format requirements and details on the produced outputs are described below.

**Table 1:** Algorithms and code sources used in MutSpec

		MutSpec-				
Packages	Version	Annot	Stat	NMF	Compare	Source
Annovar^a^	June 2015	X	-	-	-	[17]
Statistics::R^a^	0.33	-	X	-	-	http://search.cpan.org/~gmpassos/Statistics-R-0.02/lib/Statistics/R.pm#AUTHOR
Spreadsheet:: WriteExcel^a^	2.40	-	X	-	-	http://search.cpan.org/~jmcnamara/Spreadsheet-WriteExcel-2.40/lib/Spreadsheet/WriteExcel.pm
ggplot2	1.0.1	-	X	X	X	http://ggplot2.org/
gplots	2.17.0	-	X	-	-	http://cran.r-project.org/web/packages/gplots/gplots.pdf
gtable	0.1.2	-	X	-	-	http://cran.r-project.org/web/packages/gtable/gtable.pdf
reshape	1.4.1	-	X	X	X	http://cran.r-project.org/web/packages/reshape/reshape.pdf
scales	0.2.5	-	X	X	-	http://cran.r-project.org/web/packages/scales/scales.pdf
gridExtra	0.9.1	-	X	X	-	http://cran.r-project.org/web/packages/gridExtra/gridExtra.pdf
NMF	0.20.6	-	X	X	-	[18]
getopt	1.20.0	-	-	X	-	http://cran.r-project.org/web/packages/getopt/getopt.pdf
lsa	0.73.1	-	-	-	X	http://cran.r-project.org/web/packages/lsa/lsa.pdf

^a^These packages are developed in Perl while all other packages are developed in R

### Data import and formatting

The first tool to be run is MutSpec-Annot. This tool will retrieve different types of structural and functional annotations that will be used by the other MutSpec tools. MutSpec-Annot accepts variant call format (VCF, version 4.1) files as well as tab-delimited text files that may be obtained from popular sources such as the International Cancer Genome Consortium (ICGC) or The Cancer Genome Atlas (TCGA) data portals and Catalogue of Somatic mutations in Cancer (COSMIC) database. The minimal information required is, for each variant, the chromosome number, the start genomic position, the reference allele and the alternate allele. The columns containing this information should have a header. There are different supported names for these header columns (case-sensitive names) that correspond to formats of data retrieved from popular variant callers or public databases (details provided in the tool interface). These four columns may be in any order, and other columns may be present. The additional columns will be kept in the output file after the retrieved annotations. Galaxy automatically recognises file formats, but if the format of the imported files need to be corrected, it can be easily done by editing the file attribute or by using the tool Convert (a default tool available in Galaxy).

The output of MutSpec-Annot is a tab-delimited text file. This file may be used as input of the tools MutSpec-Filter and MutSpec-Split that both require a tab-delimited text file as input. These later tools are optional as the imported data may not need to be filtered or parsed (see an example analysis further below). The next tool to use is MutSpec-Stat that requires a dataset list (also named ‘collection’) as input, a specific feature of Galaxy. MutSpec-Stat is designed to calculate statistics on mutation features for each individual samples as well as on the sample pool. MutSpec-Stat generates an Excel file (see Additional file 1 as example), with results of individual samples in individual datasheets, and several html pages showing summary results for each sample with links to data downloads. MutSpec-Stat output can be used directly with the tool MutSpec-NMF for extracting mutation signatures. MutSpec-NMF also accepts a tab-delimited matrix formatted as specified in the tool interface. MutSpec-NMF output can be directly used as input of MutSpec-Compare. MutSpec-Stat, −NMF and -Compare produce results in graphical and tabular formats that can be displayed as html pages or downloaded as tab-delimited text files or images (Fig. 1).

### Annotations, filtering and databases

MutSpec-Annot uses the ANNOVAR software [17] to provide various functional annotations of variants, as well as Perl scripts developed in-house to retrieve strand orientation of transcripts and sequence context of variants. ANNOVAR includes several databases and annotation types for mouse and human genomes (required databases and corresponding genomes are listed in Table 2), among which some are optional and some are required for MutSpec tools to function properly. MutSpec has been validated for hg19 and mm9 genome builds. Other genomes and ANNOVAR databases may be installed to retrieve additional annotations or study other species based on user preferences. Database updates are regularly provided by ANNOVAR, users should thus check ANNOVAR website for these updates and install them as needed (we created an install file, listAVDB.txt that can be modified by the Galaxy administrator to specify related databases and reference genomes to be used).

**Table 2:** List of databases and reference genomes

Reference genome	Related databases	Used in
hg19	refGene	MutSpec-Stat
	genomicSuperDups	MutSpec-Filter
	snp138	MutSpec-Filter
	snp138NonFlagged	MutSpec-Filter
	1000 genome (ALL)	MutSpec-Filter
	esp6500siv2 (ALL)	MutSpec-Filter
mm9	refGene	MutSpec-Stat
	genomicSuperDups	MutSpec-Filter
	snp128	MutSpec-Filter

Once variants are annotated, the MutSpec-Filter tool may be used to filter out variants that are likely neutral polymorphisms or that are contained in duplicated regions of the genome. For the human genome, there are currently three databases available for polymorphisms: dbSNP (http://www.ncbi.nlm.nih.gov/SNP/), the 1000 genomes project (http://www.1000genomes.org/) and the Exome Sequencing Project (ESP, http://evs.gs.washington.edu/EVS/) databases. Users may filter against all three or any of these databases. Filtering against dbSNP database will remove all variants with an rs number (SNP ID). It is important to note that ANNOVAR provides two versions of dbSNP, dbSNP138 that includes all variants present in dbSNP and dbSNPNonFlagged that includes only variants that are frequent in human populations (>1 %) and that are not flagged as “clinically associated” in dbSNP database. We prefer to use dbSNPNonFlagged but users should decide. Another caution about filtering with dbSNP concerns the fact that rs numbers in dbSNP database may correspond to several variants. Although ANNOVAR will only identify exact match by taking into account not only position but also nucleotide change, annotations about a specific variant may not be accurate (more details in: “Assigning dbSNP Identifiers” at http://annovar.openbioinformatics.org/en/latest/articles/dbSNP). With 1000 genomes and ESP filters, the tool will remove variants according to a predefined standard frequency. To use different frequencies, it is recommended to use other tools proposed in Galaxy. For the mouse genome, there is currently only one SNP database available, dbSNP.

### Statistics on variant features

MutSpec-Stat provides various statistics on the characteristics of mutations observed in a human or mouse sample or group of samples (see Table 3). Briefly, summary statistics include counts and distributions of overall mutation types (six types of SBS and indels) and their functional impact (based on RefSeq annotations); the distribution of SBS in different genomic regions or by chromosome; counts and distributions of SBS in their trinucleotide sequence context (96 mutation types) and calculated on the genome sequence or only on transcribed sequences (stranded analysis). The stranded analysis calculates the strand bias for both the 6 and 96 types of SBS. Statistical tests are applied for the stranded analysis (the significance of the differences between the mutational frequencies on the non-transcribed and the transcribed strand is assessed using a chi-squared test followed by the Benjamini-Hochberg procedure for multiple testing corrections), and for the chromosomal distribution of mutations (a Pearson correlation coefficient is calculated to assess the correlation between SBS counts and chromosome size). An option in MutSpec-Stat is to compute statistics that can be used to estimate the number of mutation signatures present in the analysed dataset (an NMF R package is used [18], see next section). Another option available is the computation of statistics on the pooled samples.

**Table 3:** Analyses performed by the tool MutSpec-Stat

Analysis	Table	Graph	Statistics
Overall mutation distribution	-	X	-
Impact on protein sequence	X	X	-
SBS type distribution	X	X	-
Stranded analysis of SBS type distribution	X	X	Chi-squared test
SBS distribution by functional region	X	-	-
Strand bias by functional region	X	-	-
SBS distribution per chromosome	X	-	Pearson Correlation
Trinucleotide sequence context of SBS on the genomic sequence	X	X	-
Stranded analysis of trinucleotide sequence context of SBS	X	X	-

The output of MutSpec-Stat is an html page that contains links to summary results for each individual sample and to an Excel file (called “Report”) that contains sample datasheets with all results displayed in various formats (tables, heatmaps, bar graphs, matrices, WebLogo). Each datasheet is named after the sample ID. It is of note that because Excel has a limitation on datasheet names, sample identifiers must be within a limit of 31 characters. All individual tables and graphs can also be downloaded as individual files in a compressed archive.

### Extraction of mutation signatures

MutSpec-NMF extracts the minimal set of mutation signatures that optimally explains the proportion of each mutation type (96 types represented by the 6 base substitutions in their trinucleotide sequence context) found in each sample and then estimates the contribution of each signature to each sample. MutSpec-NMF uses the non-negative matrix factorization algorithm from Brunet et al. [19] implemented in the NMF R package developed by Gaujoux and Seoighe [18]. The Brunet algorithm has been successfully used to extract mutation signatures from somatic mutation data in human cancers [3, 8]. Here we use the default algorithm of Brunet with the Kullback–Leibler divergence penalty and a number of iterations set to 200 in order to achieve stability of the results. The aim of the method is to reduce the dimension of the original data, with the caveat that the factorisation rank needs to be specified. It is thus necessary to first estimate the factorisation rank (number of expected signatures) for the analysed dataset. This can be done with the option available in MutSpec-Stat that performs NMF with different rank values (2 to 8 by default) and compute some quality measures of the results, including the cophenic coefficient and the rss curve. The NMF R package cited above is also used to perform these analyses. The best rank value indicated by the quality measures may be used as the number of expected signatures in MutSpec-NMF as suggested by the authors of the NMF package [18]. The calculation of these statistics is optional in MutSpec-Stat because running NMF on a large dataset requires intensive computations (for estimating the rank value a total of 50 runs are performed for each value while 200 runs are performed for the full analysis). For reducing the computation time it is recommended to use all available central processing unit (CPU) on the machine where Galaxy is installed (to be checked with the Galaxy administrator). The input matrices for NMF are extracted from the output of MutSpec-Stat, so that users can select a MutSpec-Stat report as input. Users may alternatively use matrices imported from other analyses as long as they are in the required format (the tool works with a tab-delimited text file or a MutSpec-Stat report as input). For example, matrices obtained from different MutSpec-Stat analyses may be combined to run NMF on groups of samples that have been analysed separately (see example in Additional file 2).

Results are shown graphically as bar charts representing the obtained signatures or showing the contribution of each signature to the mutation load of each sample (Fig. 1). This package also performs unsupervised hierarchical clustering of samples based on mutation signature contributions. It should be noted that mutation signatures are based on 96 SBS types, the current standard in the field that is used in the COSMIC database. It does not allow deriving other types of signatures as it is designed to produce signatures comparable to the ones compiled in the COSMIC database and to produce preformatted graphs.

### Comparison of obtained signatures with published signatures

MutSpec-Compare computes the similarity between the signatures identified by MutSpec-NMF and a set of published signatures using the cosine similarity method implemented in the LSAfun R package [20]. This method measures the similarity between two vectors of an inner product space by calculating the cosine of the angle between them. The resulting values range between 0 and 1, corresponding respectively to an absence or a complete similarity. Results are displayed graphically as a heatmap and provided as a tabular matrix. A cosine value above 0.9 can be considered as a good match. For the reference signatures, user may select the matrix provided with the tool or their own matrix. The matrix provided includes 30 signatures published in the COSMIC database (v72) [21] plus four experimental signatures (methylnitronitrosoguanidine, aristolochic acid, benzo(a)pyrene, activation-induced cytidine deaminase) previously published in Olivier et al. [8]. As this tool requires two text-tabulated matrices, users may also input matrices produced by other tools as long as they are in the required format.

## Results and discussion

To illustrate MutSpec functionalities and show an example of analysis workflow, we have analysed a public dataset reporting mutation data on 106 cases of oral squamous cell carcinomas (OSCC) from India [22]. The aetiology of OSCC is linked to several risk factors, including tobacco smoking, tobacco chewing, alcohol drinking, HPV infection and UV radiations. These risk factors vary between different geographical regions, with tobacco chewing being prevalent in the Indian population while the association with tobacco smoking and alcohol drinking play major roles in Western countries. Tobacco and UV are strong mutagens that create specific types of DNA damage; one can thus expect to identify various mutation signatures reflecting exposure to these mutagens in the selected dataset. Screenshots of the following steps of analyses are provided in Additional file 3 to illustrate MutSpec functionalities.

### OSCC data retrieval and annotation

Somatic mutation data were retrieved from the ICGC data portal (ORCA-IN dataset, downloaded on June 2015). The dataset was available as a tab-delimited text file containing a single list of mutations for the 106 samples. This list was uploaded in a Galaxy history. ICGC formatted files are supported by MutSpec-Annot so no further formatting was needed.

The first step was to annotate the file with MutSpec-Annot. Variants in OSCC-IN dataset were mapped to the genome build hg19, we thus selected “hg19” as reference genome. Another option to specify is the length of the sequence context to retrieve; here we chose 1 as we are only interested in the trinucleotide sequence context (one base on each side of the variant base). One output file, OSCC-IN_annotated was thus created and appeared in the history. We then proceeded to the next step, which is to filter out potential polymorphisms with MutSpec-Filter. We filtered against all databases but dbSNP. Although the data analysed have been curated and thus should have been already filtered, 673 (5 %) variants were removed. Then, we used MutSpec-split to parse the file into individual sample files using the sample ID column. MutSpec-split automatically creates a collection of files, so we obtained a dataset collection of 106 files.

### Mutation spectra in OSCC

**Fig. 2:**
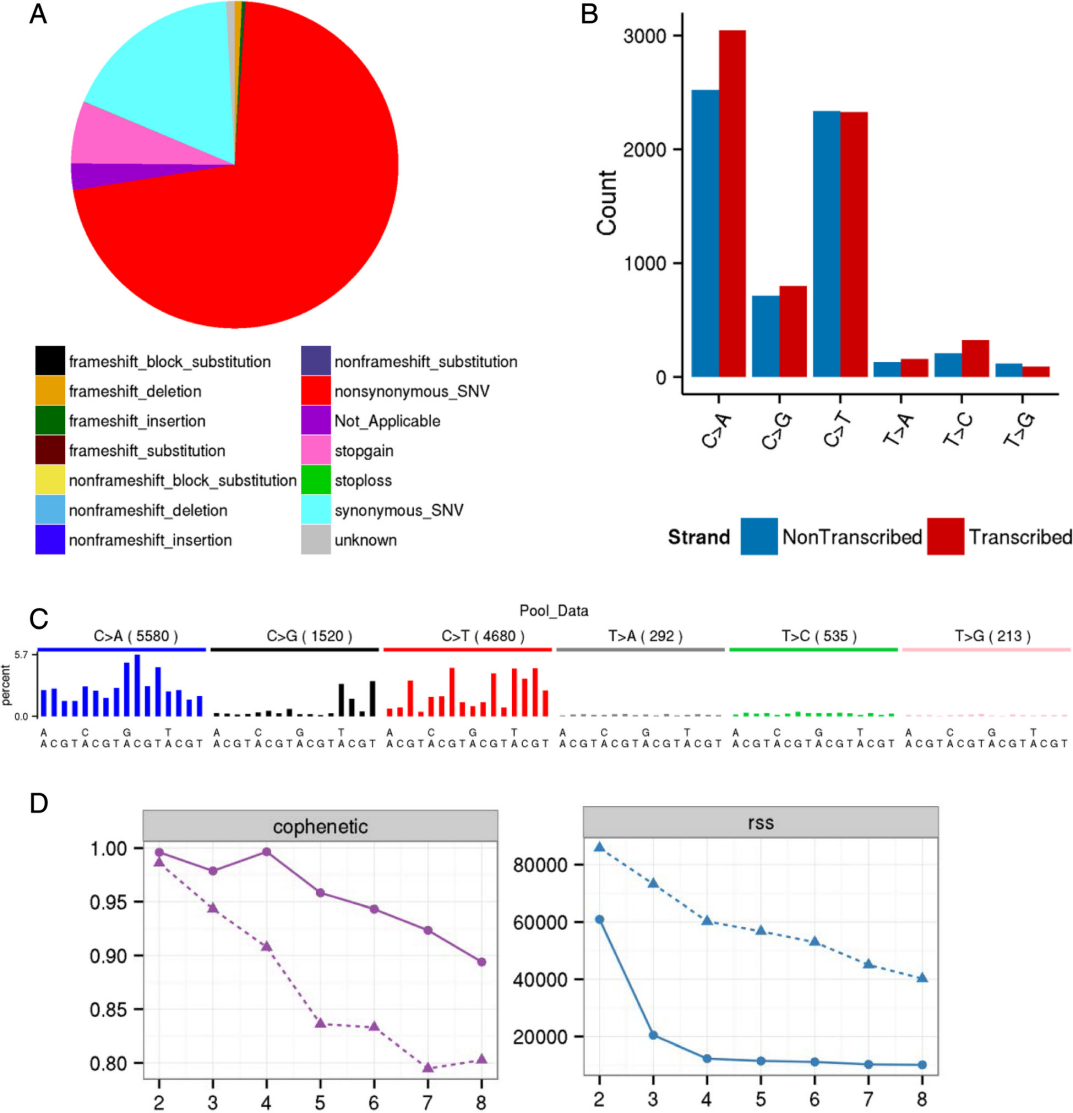
Mutation spectra in OSCC from Indian patients. Results for the pool of 106 samples are shown. **a** Distribution of variants (N = 13059) according to their functional impact on protein sequences. **b** Stranded analysis of the 6 types of SBS showing counts for SBS with transcript annotations (N = 12789). **c** Distribution of SBS according to their trinucleotide sequence context (SBS counts are indicated in parenthesis). **d** Plots of the cophenic and rss analyses using a range of factorisation values (2 to 8). The solid lines represent the results obtained with the original data while the dotted lines represent the results obtained with randomized data (original data are shuffled)

The dataset collection created by MutSpec-Split was then used as input for MutSpec-Stat to generate statistics on mutation spectra for each sample and to compute the mutation matrix to be used for extracting mutation signatures. We ran the tool with the “pool sample” option in order to obtain statistics for the pooled samples. The reference genome should be specified again at this step. Finally, we also selected the option that calculates statistics for estimating the number of signatures present in the dataset. A summary of the results are shown in Fig. 2 for the sample pool (see detailed results in Additional file 1). The overall mutation pattern shows that the majority of variants are non-synonymous SBS (Fig. 2a), and that the most frequent SBS types are C:G > A:T followed by C:G > T:A (Fig. 2b). The trinucleotide sequence context distribution of these mutations show specific patterns, with C > A occurring preferentially within 5’-GCN-3’ motifs and C > T within CpG sites (Fig. 2c). The third most frequent SBS are C > G. Both C > G and C > T occur preferentially within 5’-TCN-3’ motifs, suggesting the presence of APOBEC-induced mutations [23]. Based on the cophenic and rss statistics calculated for estimating the NMF factorization value, 4 signatures may be present in this dataset as it is the first value for which the cophenetic coefficient starts decreasing and where the rss curve presents an inflection point (Fig. 2d).

### Extraction and identification of mutation signatures in oral cancers

**Fig. 3:**
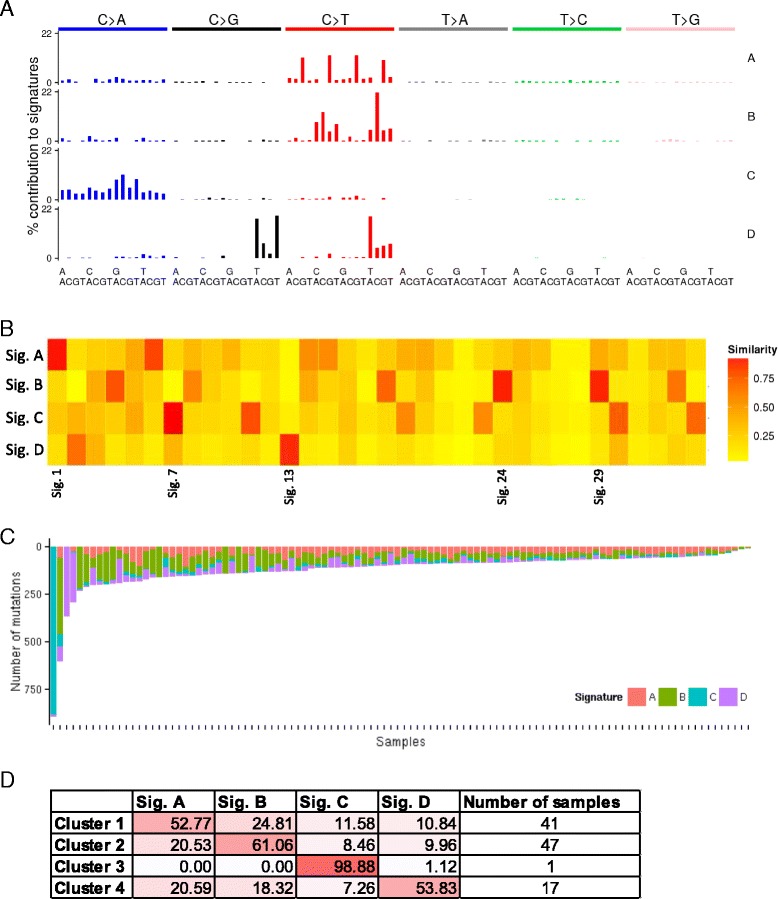
Mutation signatures in Indian OSCC and their suspected origin. Summary results of MutSpec-NMF and MutSpec-Compare analyses obtained on the 106 OSCC samples. **a** Mutation signatures obtained by NMF with a factorisation value of four. **b** Comparison of the four OSCC signatures (vertical axis) with 34 reference signatures (horizontal axis) using the cosine similarity method. The heatmap is color-coded according to the cosine value that ranges from 0 to 1. Only reference signatures with a significant match (cosine > 0.9) are labelled. **c** Number of mutations contributing to each of the four signatures identified, for each sample analyzed. **d** Average contributions of the four identified signatures to the mutation load of clustered samples and number of samples by cluster

To analyse mutation signatures present in the dataset, we ran MutSpec-NMF using the output report of MutSpec-Stat and factorisation value was set to 4. We then compared the obtained signatures with published signatures using the tool MutSpec-Compare. Fig. 3a shows the 4 signatures obtained. Signature A matched best with signature 1 (Age), signature B with signatures 29 (tobacco chewing) and 24 (aflatoxin), signature C with signature 7 (UV) and signature D with signature 13 (APOBEC) (Fig. 3b). MutSpec-NMF also produces a graph showing the total number of SBS per sample and the proportion contributing to the 4 signatures (Fig. 3c). On this graph, one sample is standing out with the largest number of SBS and a close to 100 % contribution to the UV signature (sig 7). Finally, NMF clusters samples based on their signatures composition. From these data, MutSpec-NMF produces a summary analysis that shows the number of samples by cluster and the average contributions of each signature in each cluster (Fig. 3d). In the 106 OSCC samples analysed here, we found one sample likely to be related to UV exposure (high number of SBS corresponding to the previously reported UV signature, sig.7) while a majority of samples (N = 47) had a predominant signature related to tobacco chewing and/or aflatoxin. Another large set of samples (N = 41) had the age signature as the predominant signature, and in a small number of samples (N = 17) the APOBEC signature was the most prominent. Because the cases analysed are from India where tobacco chewing is one major risk factor for oral cancer, it is more likely that the signature B found here is related to tobacco chewing and not aflatoxin. These two signatures (24 and 29) are in fact very close (they share several predominant C > A in specific contexts due to similar mechanisms of the suspected underlying carcinogens) and thus difficult to separate by NMF [5]. The fact that a majority of samples were found to carry this signature confirms the major role of tobacco chewing in the etiology of OSCC in India.

It should be noted that the NMF algorithm used in MutSpec is not expected to give identical results to that used by Alexandrov et al. which uses a complex sequence of pre-filtering and bootstrapping in addition to the NMF algorithm itself. However, certain features of the algorithm used by Alexandrov et al. do not scale with sample size (in particular setting all counts of less than 5 to zero) and are therefore not well adapted to the small sample sizes found in experimental studies or small to medium scale analyses for which MutSpec is designed.

### Performance

MutSpec may be used to analyse data from whole-exome, whole-genome or targeted sequencing experiments performed on human or mouse genomes. The tool manages CPU usage to optimize performance in term of analysis time. For example, to annotate a file with less than 5000 variants it will use one CPU, while for a file with more than 100,000 variants it will use the maximal capacity allowed by the Galaxy server administrator. For such a file, using 24 CPU will take about 7 min, using 8 cores will take about 14 min to annotate while it would take 4 h with only one CPU. Computation of the statistics for estimating the NMF factorisation value (option in MutSpec-Stat) and for running MutSpec-NMF is also time consuming when large number of samples are analysed. Here the tool will use the maximum allowed CPU capacity.

There is no limit in the number of samples or mutation per sample that can be analysed. However, the capacity to open Excel files with large number of datasheets (over 500) will depend on user’s computer settings and performance (ie, a file with 530 samples takes 18 s to open up on a computer with 2 GB of RAM). This limitation can be overcome by adapting the design of the analysis workflow to limit the number of samples included in one Excel file. All graphical outputs generated by the tools can be downloaded as high resolution images suitable for publication (300 dpi). MutSpec toolbox is well documented with short descriptions of input and output formats and options for each tool. While the methods for defining and extracting mutation signatures implemented in MutSpec correspond to current standards well accepted in the field, we will provide package updates and upgrades reflecting progresses in the field, such as new format or definition for mutation signatures.

## Conclusions

MutSpec offers an easy-to-use framework for variant annotation and statistical analyses of mutation patterns from genome-wide data obtained from deep sequencing experiments. It is based on the Galaxy open-source framework that offers a powerful management system for reproducible bioinformatics analyses. MutSpec accepts the standard VCF format as input as well as any list of variants in tab-delimited text format and implements established methods. The example analysis presented here illustrates the straightforward workflow and the richness of the statistics and publication-grade graphics produced by the tool. MutSpec is versatile as data from both human and mouse and from different genome builds can be analysed for easy comparison of human and experimental data. To our knowledge, MutSpec is the only tool available as a graphical interface to researchers with no computer programing skills or higher level of bioinformatics expertise in the analysis of mutation signatures present in cancer genomes. Given the positive feedbacks from test users, we believe that MutSpec can be a very useful tool for a large community in the field of genomics, namely investigators interested in interpreting the mutational processes that contribute to human carcinogenesis.

## Availability and requirements

Project name: MutSpec

Availability: MutSpec package in the Galaxy toolshed at https://toolshed.g2.bx.psu.edu/

Operating system(s): Linux.

Programming language: Perl (version 5.18.1), R (version 3.1.2), XML, HTML

Other requirements: Galaxy, ANNOVAR

License: GPLv2

## Additional files

Additional file 1: Example of Excel file generated by MutSpec-Stat tool. The data in this file correspond to the example analysis of OSCC described herein. (XLS 37674 kb) Additional file 2: Example of NMF analysis with combined matrices from different analyses. Matrices from two different analyses may be combined in a single matrix to analyse samples from analysis 1 and 2 together. This matrix should contain a header with sample IDs and have 96 rows describing the 6 SBS types in their sequence context. The matrix should be formatted as tab-delimited text to be accepted as input of MutSpec-NMF. (PPT 348 kb) Additional file 3: Screenshots of MutSpec tools inputs and outputs in Galaxy. (PPT 2468 kb)

## Abbreviations

COSMIC: Catalogue of Somatic Mutations in Cancer
CPU: Central Processing Unit
ESP: Exome Sequencing Project
ICGC: International Cancer Genome Consortium
NMF: Non-negative Matrix Factorisation
OSCC: Oral Squamous Cell Carcinomas
SBS: Single Base Substitution
SNP: Single-Nucleotide Polymorphism
TCGA: The Cancer Genome Atlas
VCF: Variant Call Format
