# Supplementary material for: MutSpec: a Galaxy toolbox for streamlined analyses of somatic mutation spectra in human and mouse cancer genomes
Source: BMC Bioinformatics. 2016 Apr 18;17:170. doi: 10.1186/s12859-016-1011-z (PMC4835840; doi:10.1186/s12859-016-1011-z)
Supplement: Additional file 2: — Example of NMF analysis with combined matrices from different analyses. Matrices from two different analyses may be combined in a single matrix to analyse samples from analysis 1 and 2 together. This matrix should contain a header with sample IDs and have 96 rows describing the 6 SBS types in their sequence context. The matrix should be formatted as tab-delimited text to be accepted as input of MutSpec-NMF. (PPT 348 kb) [file 12859_2016_1011_MOESM2_ESM.ppt]

## Slide 1
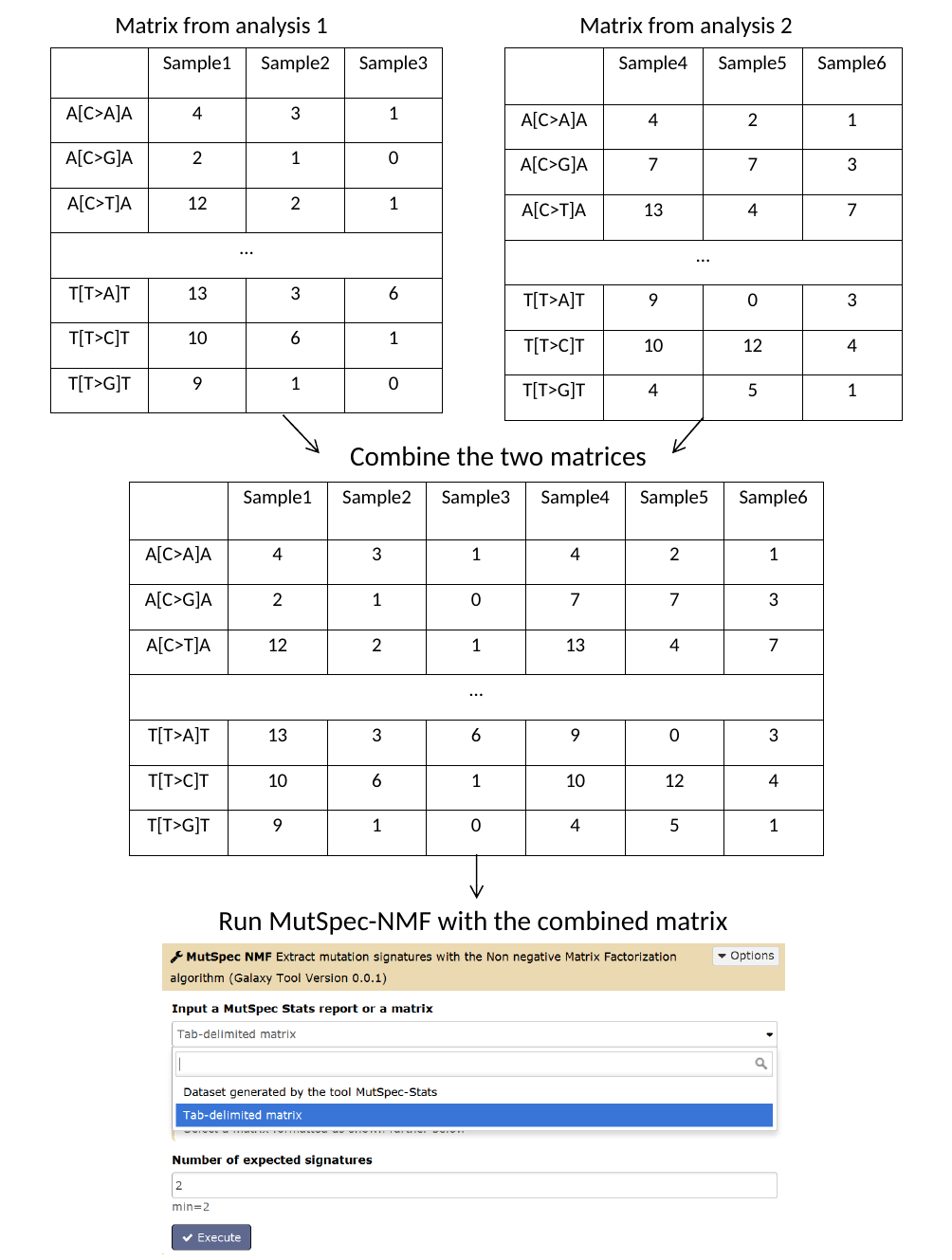

Matrix from analysis 1
Matrix from analysis 2
| | Sample1 | Sample2 | Sample3 |
| --- | --- | --- | --- |
| A[C>A]A | 4 | 3 | 1 |
| A[C>G]A | 2 | 1 | 0 |
| A[C>T]A | 12 | 2 | 1 |
| … | | | |
| T[T>A]T | 13 | 3 | 6 |
| T[T>C]T | 10 | 6 | 1 |
| T[T>G]T | 9 | 1 | 0 |
| | Sample4 | Sample5 | Sample6 |
| --- | --- | --- | --- |
| A[C>A]A | 4 | 2 | 1 |
| A[C>G]A | 7 | 7 | 3 |
| A[C>T]A | 13 | 4 | 7 |
| … | | | |
| T[T>A]T | 9 | 0 | 3 |
| T[T>C]T | 10 | 12 | 4 |
| T[T>G]T | 4 | 5 | 1 |
Combine the two matrices
| | Sample1 | Sample2 | Sample3 | Sample4 | Sample5 | Sample6 |
| --- | --- | --- | --- | --- | --- | --- |
| A[C>A]A | 4 | 3 | 1 | 4 | 2 | 1 |
| A[C>G]A | 2 | 1 | 0 | 7 | 7 | 3 |
| A[C>T]A | 12 | 2 | 1 | 13 | 4 | 7 |
| … | | | | | | |
| T[T>A]T | 13 | 3 | 6 | 9 | 0 | 3 |
| T[T>C]T | 10 | 6 | 1 | 10 | 12 | 4 |
| T[T>G]T | 9 | 1 | 0 | 4 | 5 | 1 |
Run MutSpec-NMF with the combined matrix
