# Supplementary material for: MutSpec: a Galaxy toolbox for streamlined analyses of somatic mutation spectra in human and mouse cancer genomes
Source: BMC Bioinformatics. 2016 Apr 18;17:170. doi: 10.1186/s12859-016-1011-z (PMC4835840; doi:10.1186/s12859-016-1011-z)
Supplement: Additional file 3: — Screenshots of MutSpec tools inputs and outputs in Galaxy. (PPT 2468 kb) [file 12859_2016_1011_MOESM3_ESM.ppt]

## Slide 1
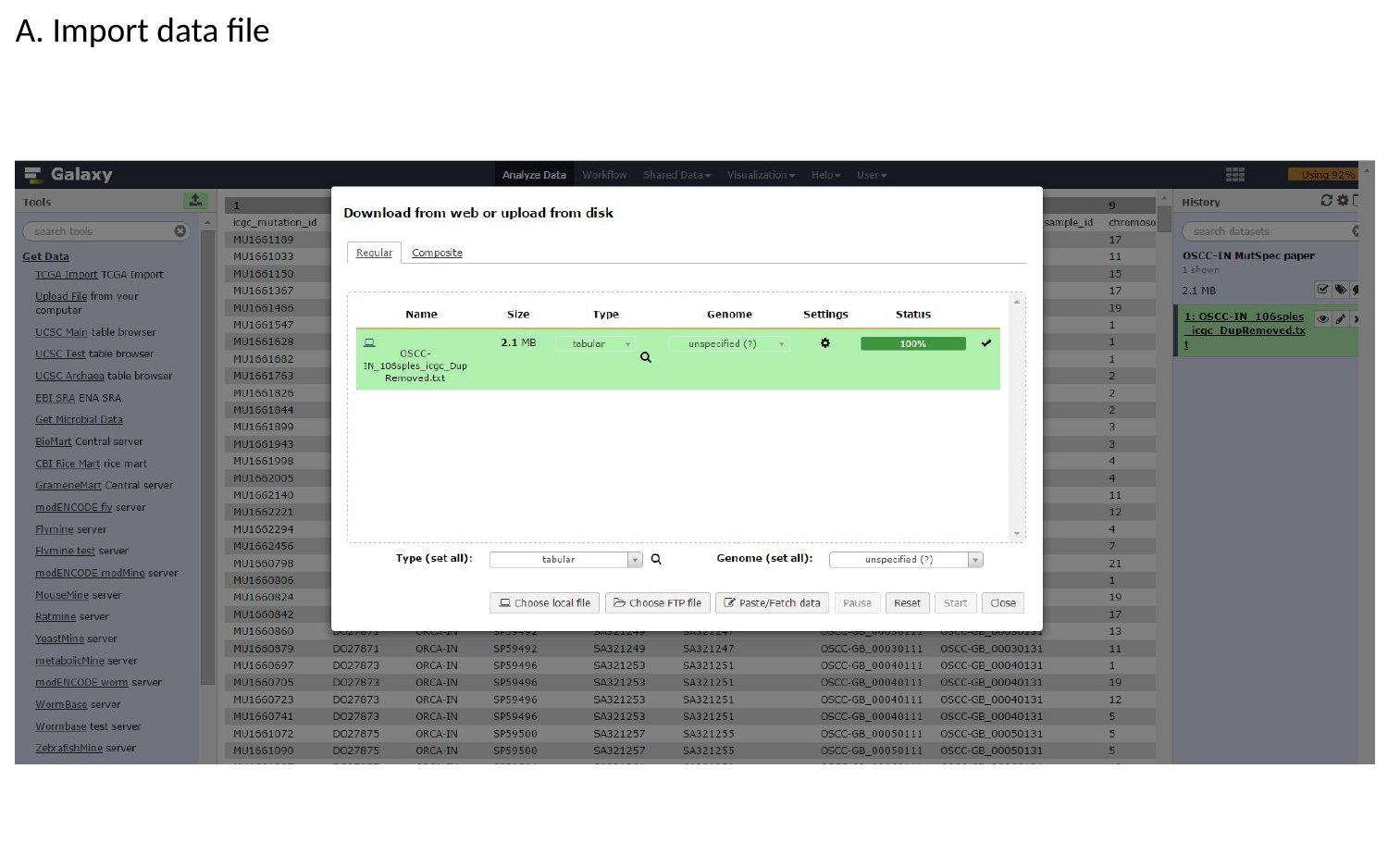

A. Import data file

## Slide 2
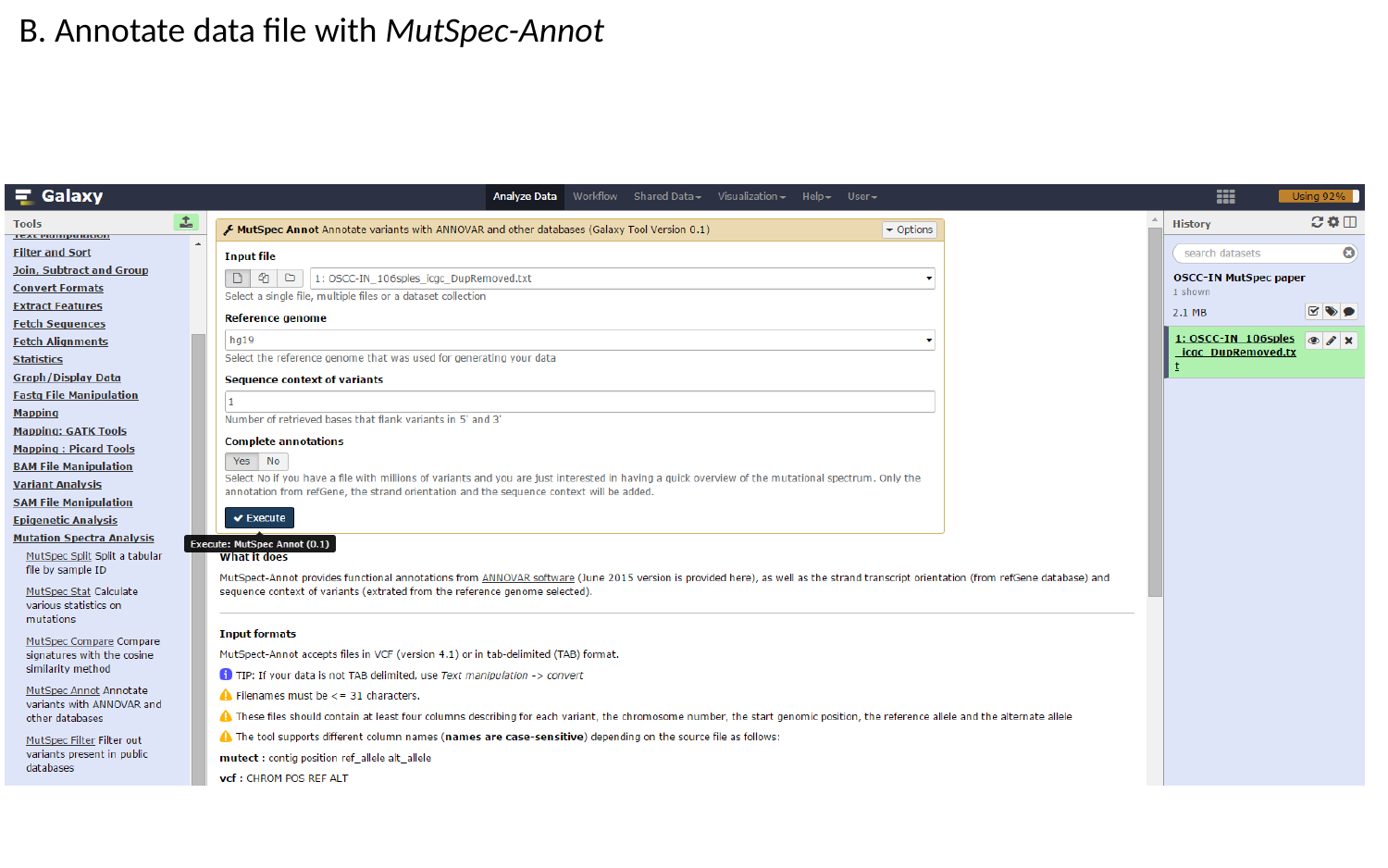

B. Annotate data file with MutSpec-Annot

## Slide 3
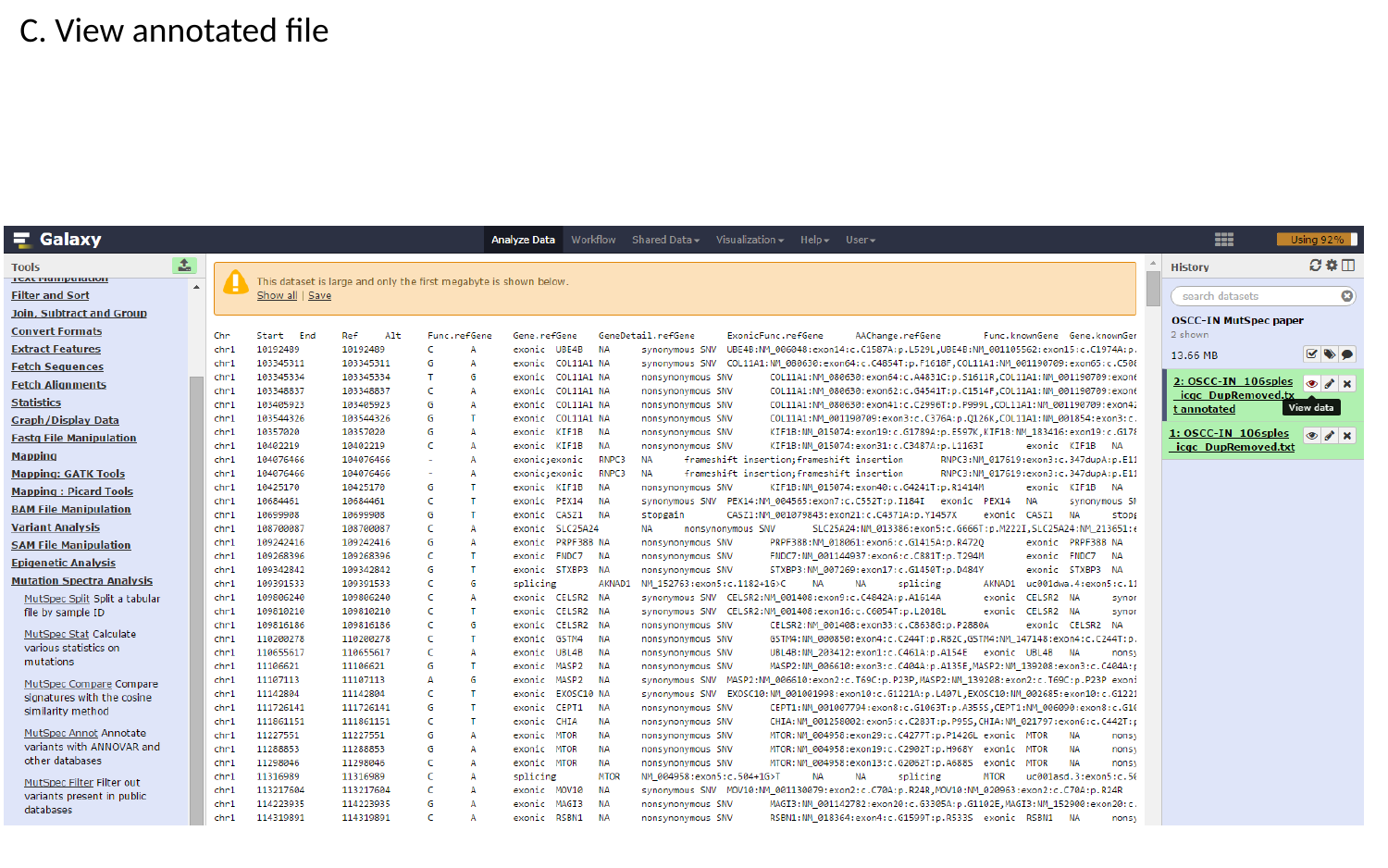

C. View annotated file

## Slide 4
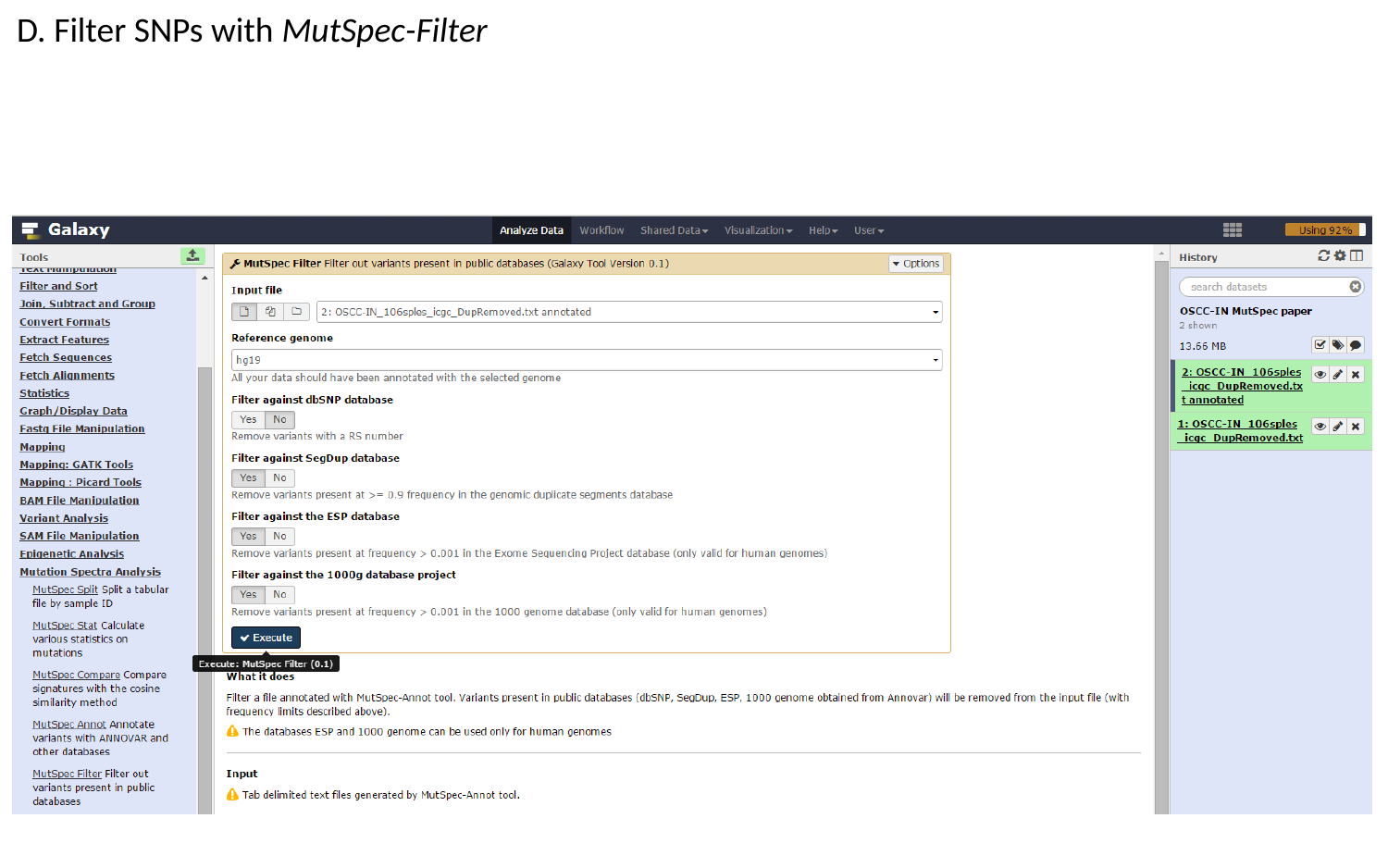

D. Filter SNPs with MutSpec-Filter

## Slide 5
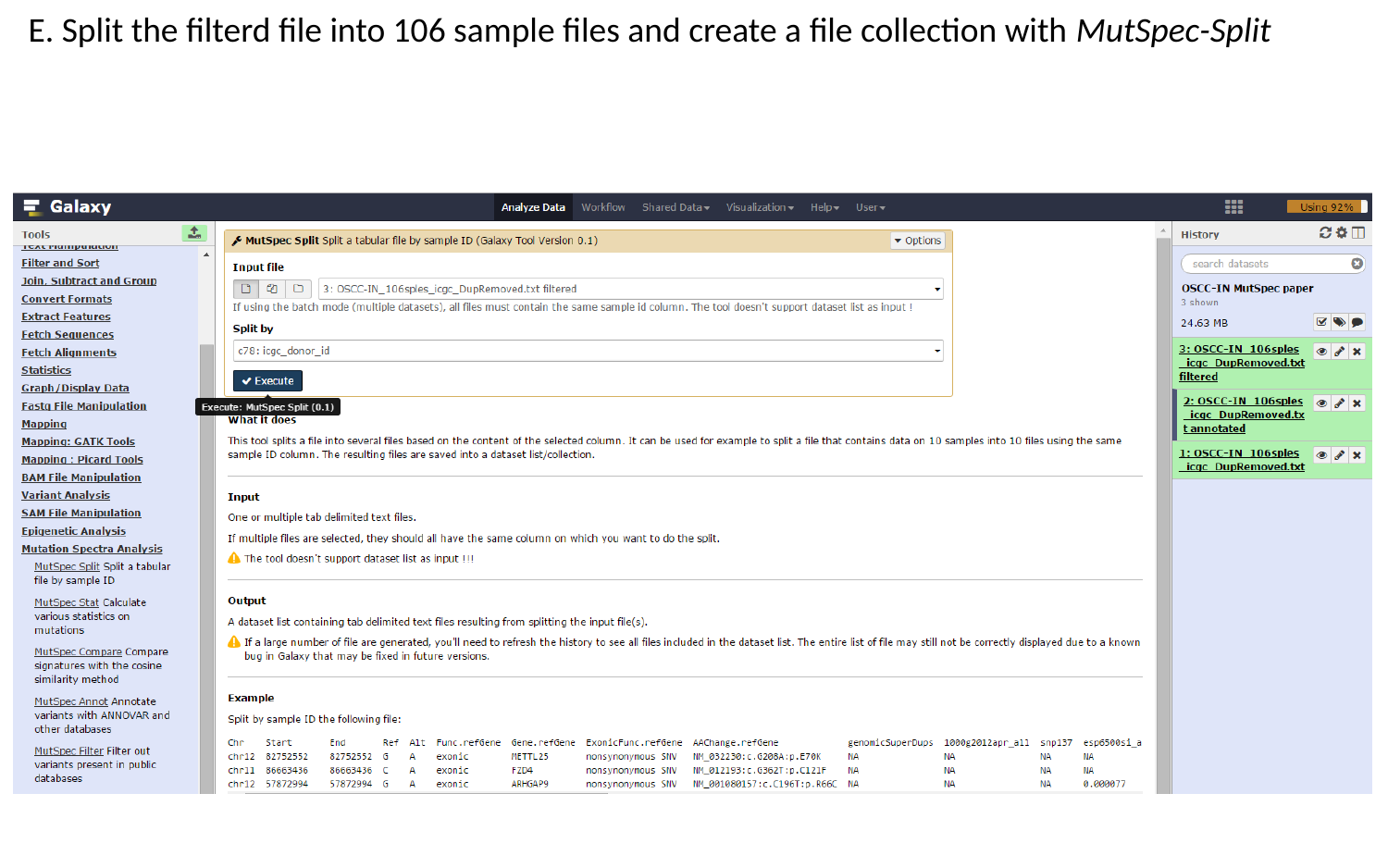

E. Split the filterd file into 106 sample files and create a file collection with MutSpec-Split

## Slide 6
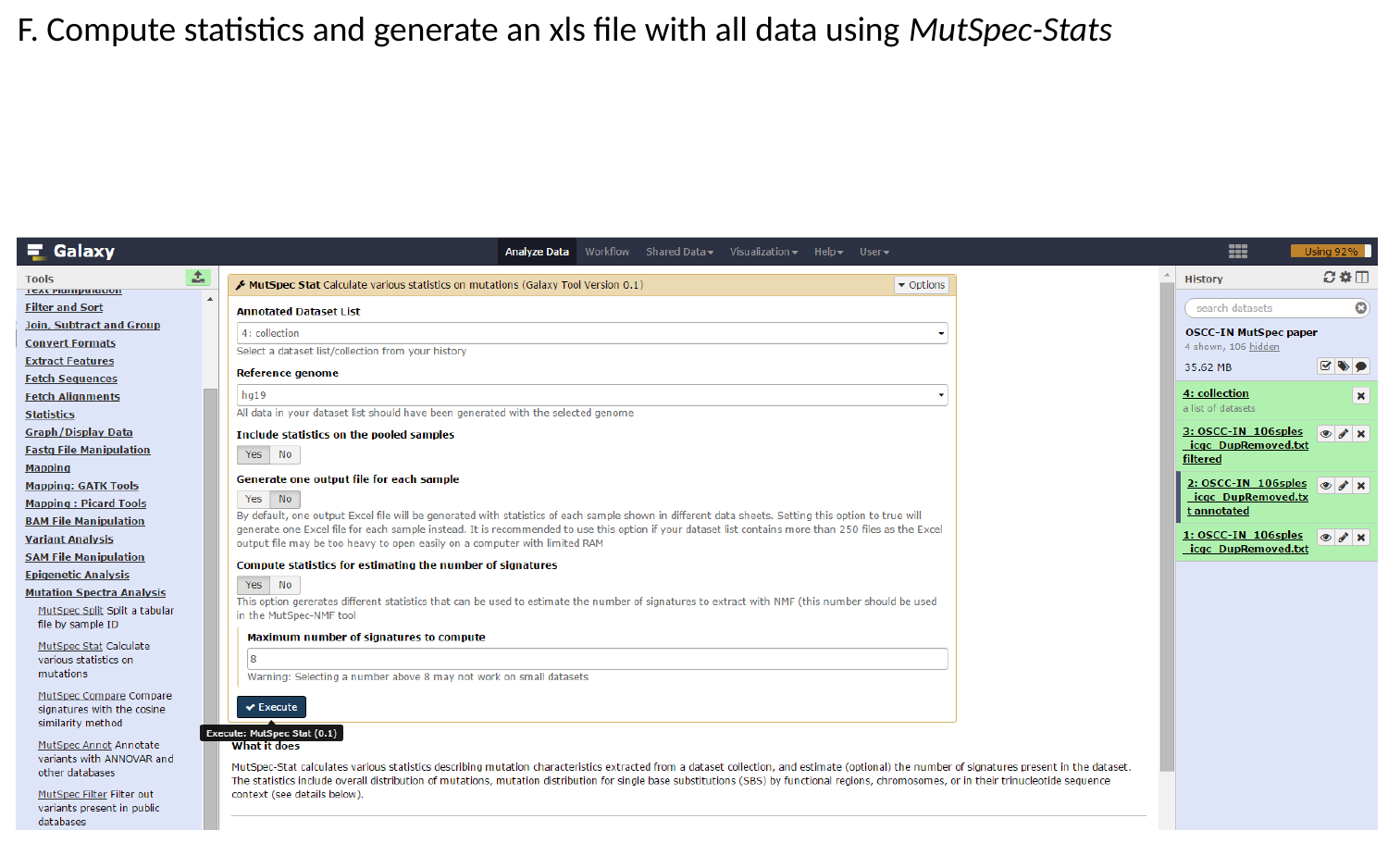

F. Compute statistics and generate an xls file with all data using MutSpec-Stats

## Slide 7
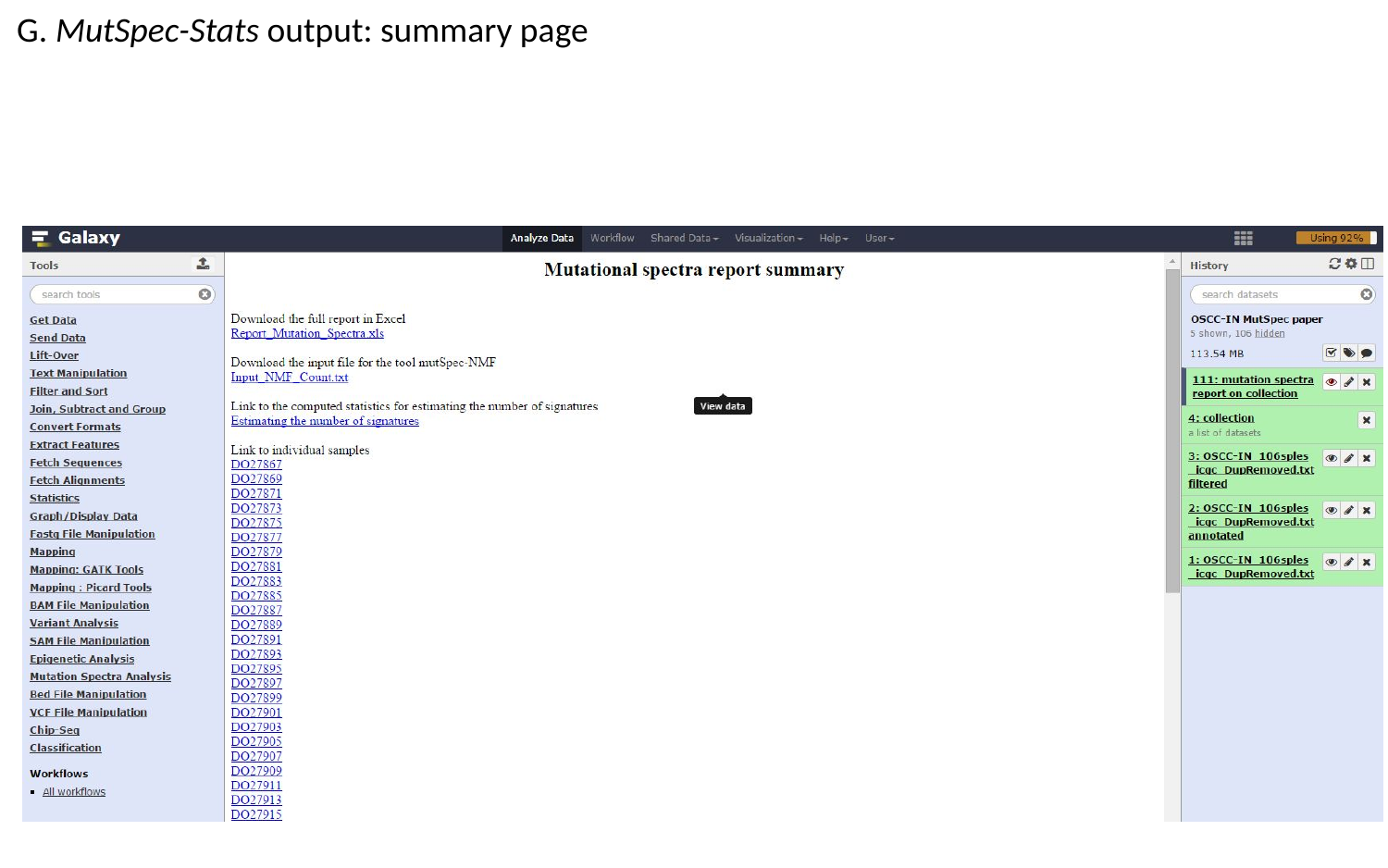

G. MutSpec-Stats output: summary page

## Slide 8
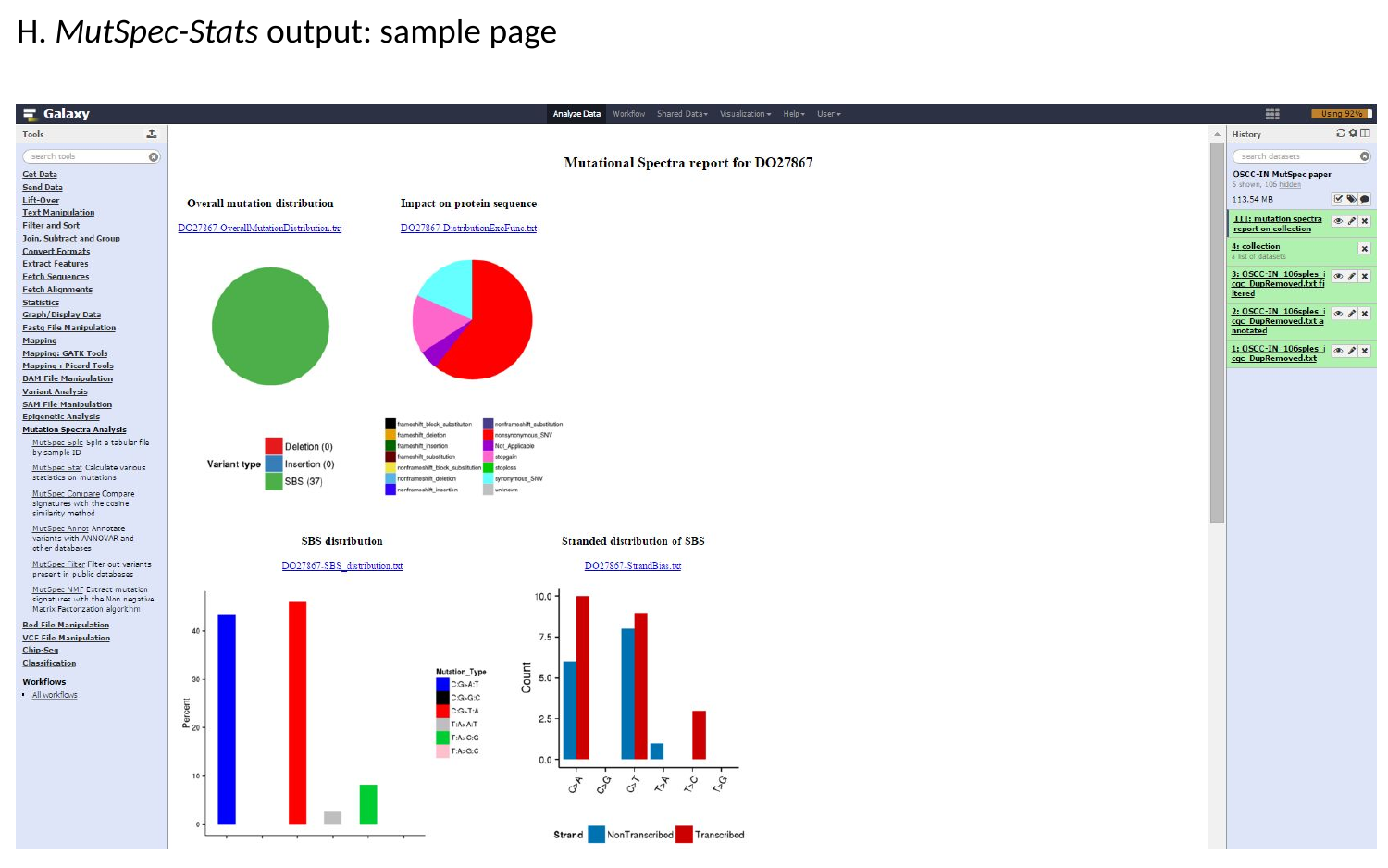

H. MutSpec-Stats output: sample page

## Slide 9
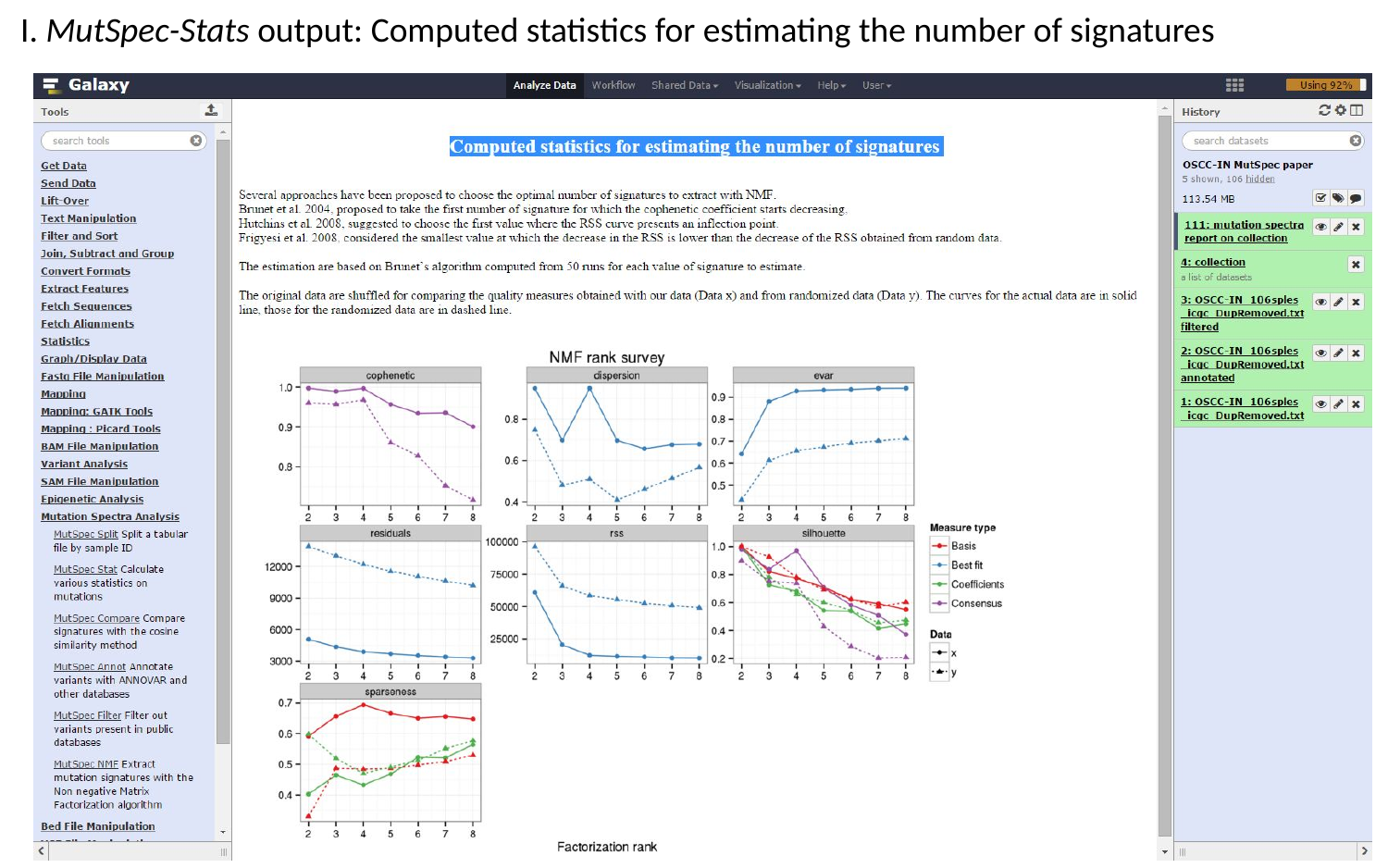

I. MutSpec-Stats output: Computed statistics for estimating the number of signatures

## Slide 10
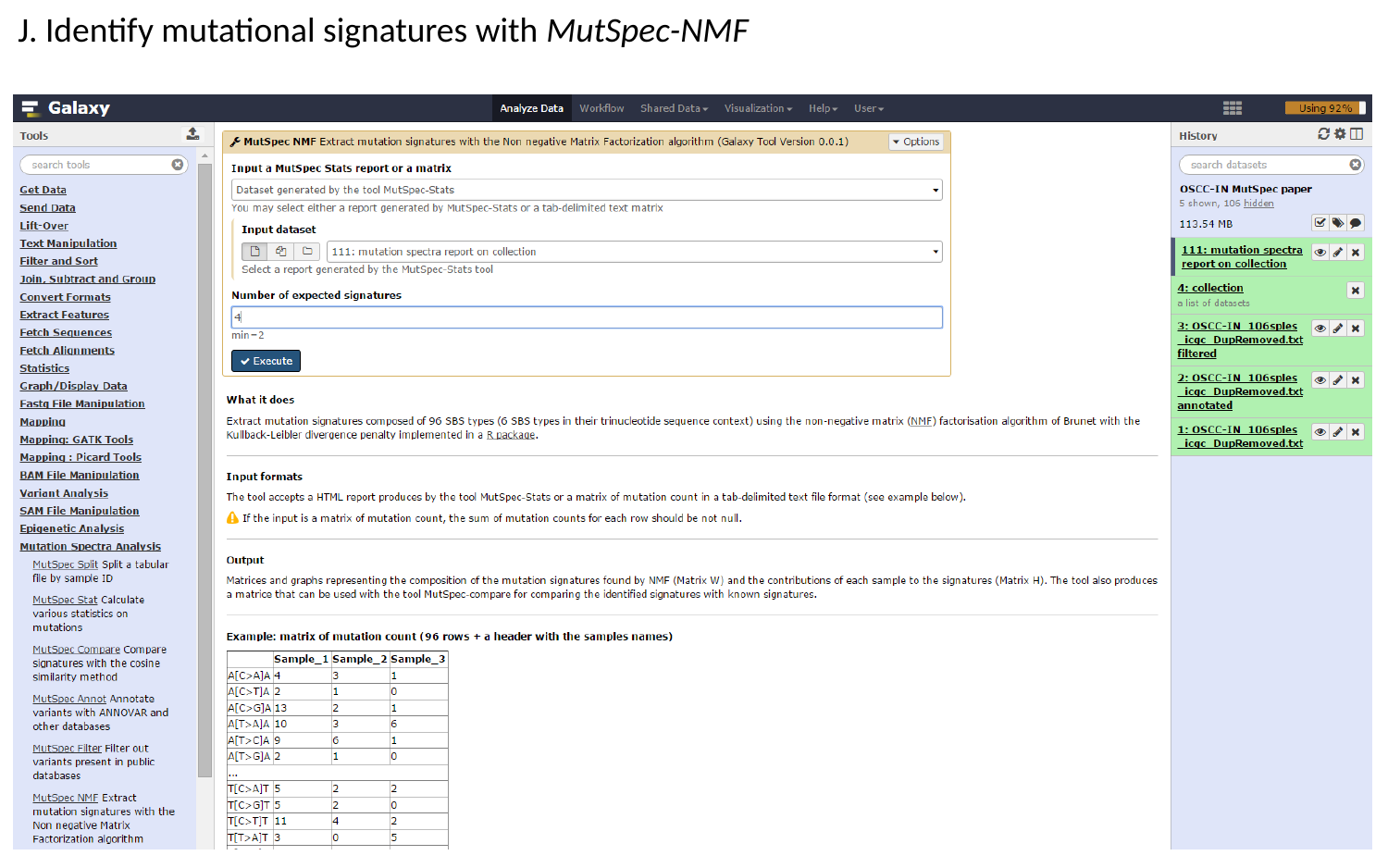

J. Identify mutational signatures with MutSpec-NMF

## Slide 11
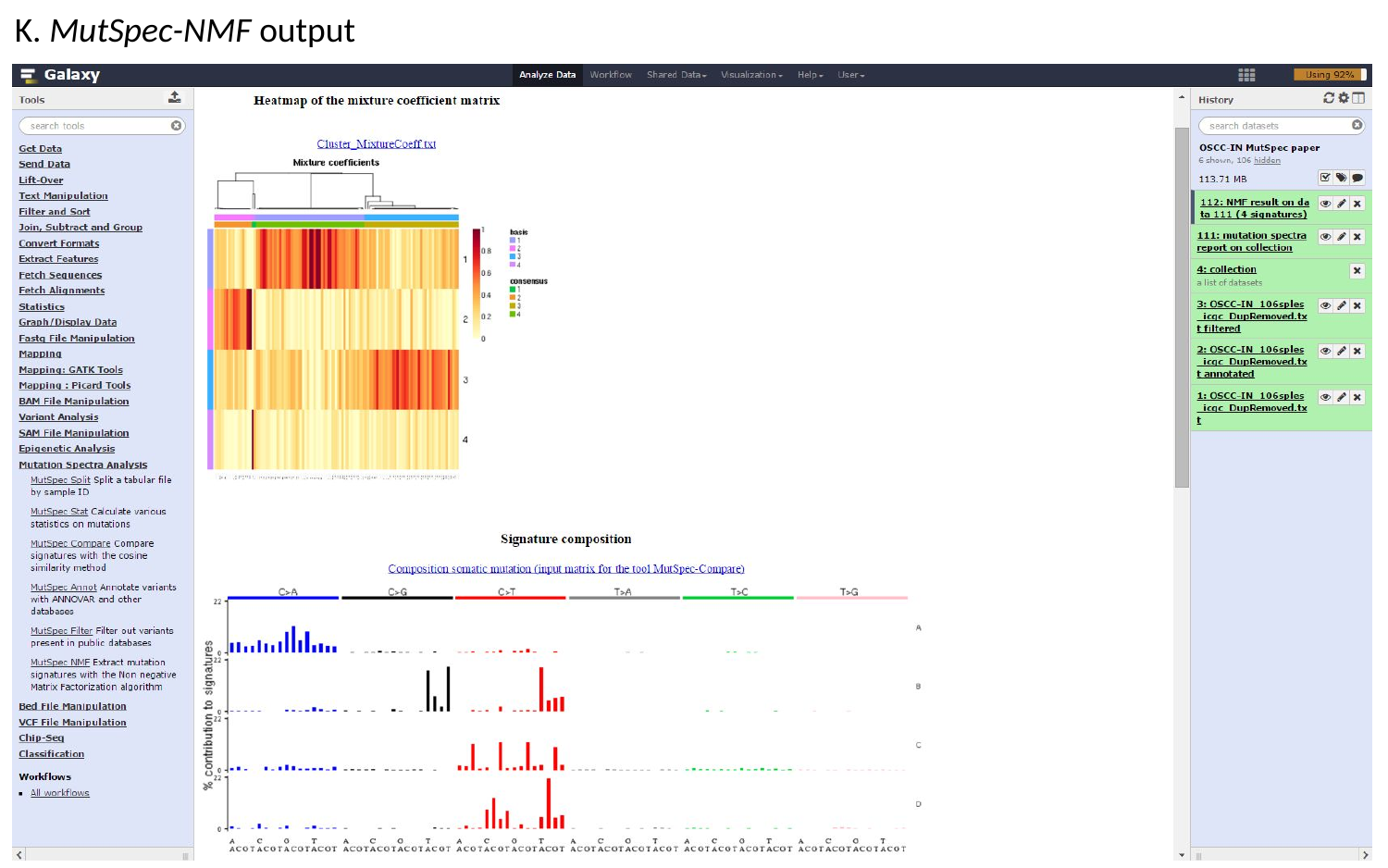

K. MutSpec-NMF output

## Slide 12
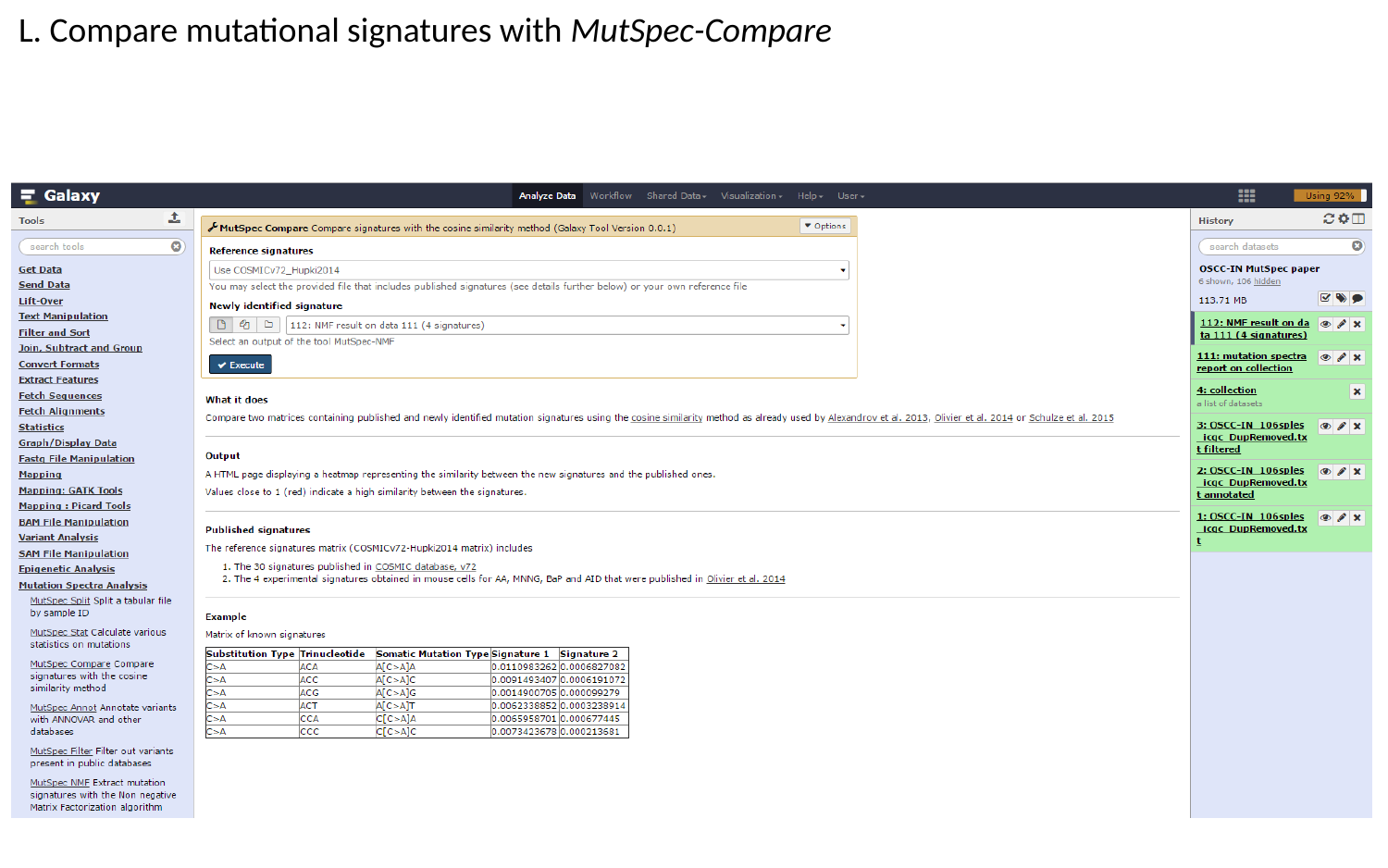

L. Compare mutational signatures with MutSpec-Compare

## Slide 13
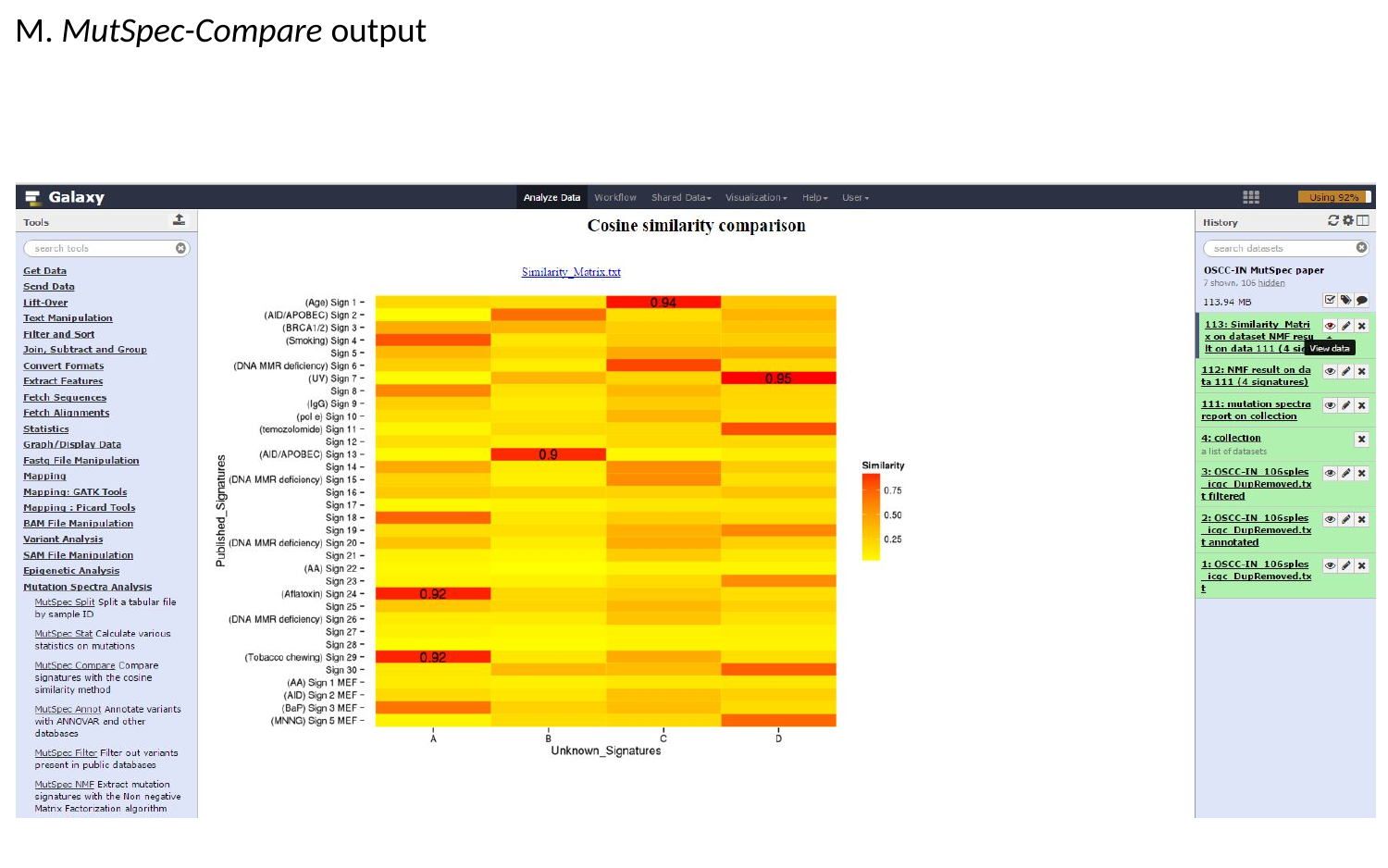

M. MutSpec-Compare output
